# Selection of hematopoietic stem cell transplantation for T-cell lymphoblastic lymphoma

**DOI:** 10.3389/fonc.2023.1193237

**Published:** 2023-07-20

**Authors:** Zhen Li, Binglei Zhang, Xinxin Fan, Ruirui Gui, Fengkuan Yu, Juan Wang, Yanli Zhang, Keshu Zhou, Yanyan Liu, Yufu Li, Jing Ding, Yongping Song, Jian Zhou

**Affiliations:** ^1^ Department of Hematology, Affiliated Cancer Hospital of Zhengzhou University and Henan Cancer Hospital, Zhengzhou, Henan, China; ^2^ Department of Hematology, The First Affiliated Hospital of Zhengzhou University, Zhengzhou, Henan, China

**Keywords:** hematopoietic stem cell transplantation, T-cell, lymphoblastic lymphoma, auto-HSCT, allo-HSCT

## Abstract

**Background:**

Hematopoietic stem cell transplantation (HSCT) is an important treatment for T-cell lymphoblastic lymphoma/leukemia (T-LBL). To compare the efficacy and influencing factors of autologous hematopoietic stem cell transplantation (auto-HSCT) with those of allogeneic hematopoietic stem cell transplantation (allo-HSCT) from different donors for the treatment of T-cell lymphoblastic lymphoma/leukemia (T-LBL) and provide a basis for selection of appropriate transplant methods and donors.

**Methods:**

To provide evidence of appropriate transplant methods for these patients, we retrospectively summarized the clinical characteristics of 75 T-LBL patients receiving HSCT at Henan Cancer Hospital between March 2012 and October 2021. Overall survival (OS), progression-free survival (PFS), cumulative incidence of relapse (CIR), non-relapse mortality (NRM), and related factors affecting efficacy were analyzed.

**Results:**

The 3-year CIR (39.9% vs 31.1%, P=0.745), 3-year PFS (60.1% vs 49.6%, P=0.434), and 3-year OS (62.8% vs 53.0%, P=0.450) were not significantly different between the auto-HSCT and allo-HSCT groups. However, the 3-year NRM was significantly higher in the allo-HSCT group (0% vs 27.2%, P=0.033). Multivariate analysis showed that the first complete remission (CR1) after HSCT was an independent influencing factor of higher OS (HR=2.498, P=0.029) and PFS (HR=2.576, P=0.016). The absence of mediastinal invasion in patients receiving HSCT was an independent influencing factor of better PFS (HR=2.977, P=0.029) and lower CIR (HR=4.040, P=0.027). With respect to the impact of donor source, the NRM in the unrelated donor (URD) and haploid donor (HPD) groups was significantly higher than that in the auto-HSCT group (P=0.021 and P=0.003, respectively), while there was no significant difference between matched sibling donors (MSD) and auto-HSCT. Compared with the MSD-HSCT group, the auto-HSCT group showed an increasing trend in 3-year CIR (39.9 ± 11.1% vs 32.6 ± 11.2%, P=0.697) and a lower trend in 3-year OS (62.8 ± 11.4% vs 64.4 ± 12.2%, P=0.929).

**Conclusions:**

HSCT is an effective consolidation treatment option for patients with T-LBL without mediastinal invasion and with CR1 before transplantation. For CR1 patients, auto-HSCT and MSD-HSCT are effective modalities for improving survival. In non-CR1 patients without an MSD, matched unrelated donors and haploidentical donor transplantations are the best treatment options to reduce relapse and improve prognosis.

## Background

T lymphoblastic lymphoma (T-LBL) is an aggressive non-Hodgkin’s lymphoma (NHL) of rare lymphoblastoid origin in the T-cell lineage. It most commonly occurs in children and young adults; predominantly in males; and often involves the mediastinum, bone marrow, and central nervous system. T-LBL progresses rapidly, converting to acute lymphoblastic leukemia (ALL) in a short time, and has a poor prognosis, with a 5-year survival rate of <20%. Intensive combination chemotherapy regimens achieve complete remission in most patients with T-LBL (1). However, the disease has a high relapse rate, and the long-term survival rate from chemotherapy alone is low. In addition, survival after relapse or treatment failure is very poor, with overall survival (OS) of 10–30%. Long-term survival has been reported for a few patients, most of whom received allogeneic transplants after salvage re-induction chemotherapy (2, 3). Although the prognosis of pediatric T-LBL has improved significantly with the continuous improvement and modification of treatment protocols, the prognostic outcome of adult T-LBL remains unsatisfactory (4). In addition, no reliable prognostic factors in adult T-LBL have been defined, as in T-ALL (1, 5). The lack of immune-targeted therapy makes hematopoietic stem cell transplantation (HSCT) the only method to prolong patient survival (6).

One study showed that first-line HSCT might be a feasible treatment option for T-LBL, even in the era of leukemia-type initial therapy (7). However, there are no uniform criteria for the exact type of transplantation used. Autologous HSCT (auto-HSCT) has rapid hematopoietic reconstitution and few adverse effects, but its application is mainly limited by the lack of graft versus-lymphoma (GVL) effect, possible contamination of tumor cells in the graft, and high relapse rate. Allogeneic HSCT (allo-HSCT) has the advantages of a GVL effect and low relapse rate, but transplantation-related complications and transplantation-related death have adverse effects on its efficacy (8). To date, the choice of transplantation and the type of transplantation for optimal survival and prognosis in patients with T-LBL remains controversial, and relevant data and guidelines are still lacking (9, 10). In this regard, a comprehensive analysis of the different disease states and risk stratification of patients is needed to provide a basis for individualized treatment plans. Therefore, this study aimed to compare the efficacy of auto-HSCT with that of allo-HSCT and matched sibling donor HSCT (MSD-HSCT) to provide evidence for future transplantation options for patients with T-LBL.

## Patients and methods

This retrospective study was approved by the Ethics Committee of Henan Cancer Hospital and was conducted according to the Declaration of Helsinki (11). All patients provided informed consent for treatment.

The study included 75 T-LBL patients who underwent peripheral blood HSCT between March 2012and October 2021 at Henan Cancer Hospital. All patients had confirmed diagnosis based on flow cytometry, cytomorphology, immunophenotype, and clinical diagnosis (10, 12). All patients were treated with chemotherapy and symptomatic support according to the relevant treatment guidelines (12–15). Chemotherapy was mainly a pediatric-like ALL treatment protocol, and efficacy was evaluated before the transplantation (16). The patients were divided into the auto-HSCT group (n=24) and allo-HSCT group (n=51). Allo-HSCT included MSDs (n=21) and alternative donors (ADs) (n=30). Of these, 20 were unrelated donors (URDs) and 10 were haploidentical donors (HPDs).

The mobilization scheme of the auto-HSCT group was bone marrow inhibitory chemotherapy combined with granulocyte colony-stimulating factor (G-CSF). The dose of G-CSF was 8–10 µg/(kg·d), and autologous peripheral blood stem cells were collected. The allo-HSCT group was mobilized with G-CSF at a dose of 5–10 µg/(kg·d). Peripheral blood stem cells from healthy donors were collected after continuous subcutaneous injection for 4–6 days. At the time of transplantation, 17 and 19 patients in the auto-HSCT group had first complete remission (CR1) and combined mediastinal invasion, respectively. Meanwhile, 42 and 30 patients in the allo-HSCT group had CR1 and combined mediastinal invasion, respectively. There were 18 and 29 patients who received total body irradiation (TBI)-based conditioning regimens, and 6 and 22 patients who received non-TBI-based conditioning regimens, in the auto-HSCT and allo-HSCT groups, respectively.

Graft-versus-host disease (GVHD) diagnosis and grading were based on the National Institutes of Health Consensus Development Projects on Criteria for Clinical Trials in Chronic GVHD and Seattle criteria (17–19). GVHD prophylaxis in the allo-HSCT group involved cyclosporine A (CsA) + mycophenolate mofetil (MMF) ± short-course methotrexate (MTX)/cyclophosphamide (CTX). The plasma concentration of CsA was assessed every 3 days and maintained at 200–400 ng/mL. All patients were provided with timely and comprehensive support for symptomatic treatment, including the prevention of infection and hemorrhagic cystitis, the use of granulocyte colony-stimulating factor, and infusion of blood products. Neutrophil engraftment time was calculated starting from the first day when the absolute value of peripheral blood neutrophil count was > 0.5×10^9^/L for 3 consecutive days. The megakaryocyte engraftment day was defined as the first day when the platelet count was >20×10^9^/L and no platelet transfusion was required for 7 consecutive days. Hematopoietic stem cell engraftment was based on polymerase chain reaction (PCR) detection of short tandem repeat (PCR-STR) genetic markers. Karyotype analysis, fluorescence *in situ* hybridization, and blood grouping were used to determine the results.

The primary endpoints were overall survival (OS), progression-free survival (PFS), and non-relapse mortality (NRM). OS was determined from the day of transplantation to any-cause death or censored at the end of follow-up. PFS was calculated from the day of transplantationto relapse, any-cause death, or the end of follow-up. NRM was defined as non-relapse death after hematopoietic stem cell engraftment. Classification data were represented as composition ratios. The count data were compared using the chi-squared or Fisher’s exact test, as appropriate. Univariate analyses of OS, PFS, cumulative incidence of relapse (CIR), and NRM were performed using the Kaplan–Meier method. The impact of factors on survival time was compared using the log-rank test. Significant risk factors in the univariate analyses (*P*<0.1) were included in a Cox regression model for multivariate analyses. All statistical analyses were performed using GraphPad Prism 8.0 (GraphPad Software, La Jolla, CA, USA) and SPSS software (version 25.0; IBM Corp., Armonk, NY, USA). All statistical tests were two tailed, and *P<*0.05 was considered statistically significant.

## Results

### Patient characteristics

The median patient age of the overall population was 24 (18–53) years; auto-HSCT group, 24 (18–53) years; and allo-HSCT group, 23 (18–49) years. There were 18 male and 6 female patients in the auto-HSCT group and 38 male and 13 female patients in the allo-HSCT group. The median numbers of transfused mononuclear cells (MNC) were 9.67 (3.75–53.13)×10^8^/kg and 11.69 (5.18–29.69)×10^8^/kg, and the median numbers of transfused CD34+ cells were 2.95 (1.65–38.82)×10^6^/kg and 5.63 (1.66–12.78)×10^6^/kg in the auto-HSCT and allo-HSCT groups, respectively. There were no significant differences in basic clinical characteristics between the auto-HSCT and allo-HSCT groups, except for the presence of bone marrow invasion (**Table 1**). In addition, we also compared the characteristics of the MSD and AD groups in the allo-HSCT group. The patients were relatively younger in the AD group, and there were no significant differences in the other characteristics (**Table 1**).

**Table 1:** Characteristics of patients between allo-HSCT and auto-HSCT groups (N=75).

Characteristics	auto-HSCT(n=24)	allo-HSCT(n=51)	*χ^2^* value	*P* value
Gender (*n*, %)			0.002	0.964
Male	18 (75.0)	38 (74.5)		
Female	6 (25.0)	13 (25.5)		
Age (year) (*n*, %)			0.567	0.451
<24 years	10 (41.7)	26 (51.0)		
≥24 years	14 (58.3)	25 (49.0)		
IPI scores (*n*, %)			2.630	0.105
0-2	17 (70.8)	26 (51.0)		
≥3	7 (29.2)	25 (49.0)		
B symptoms (*n*, %)			1.230	0.267
Yes	5 (20.8)	17(33.3)		
No	19 (79.2)	34 (66.7)		
Disease status at HSCT (*n*, %)			1.290	0.256
CR1	17 (70.8)	42 (82.4)		
non-CR1	7 (29.2)	9 (17.6)		
Invasion of the mediastinum			2.982	0.084
Yes	19 (79.2)	30 (58.8)		
No	5 (20.8)	21 (41.2)		
Invasion of the bone marrow			32.625	0.001
Yes	6 (25.0)	46 (90.2)		
No	18 (75.0)	5 (9.8)		
Conditioning regimen			2.295	0.130
TBI-based	18 (75.0)	29 (56.9)		
Non-TBI-based	6 (25.0)	22 (43.1)		
Relapse (*n*, %)			1.580	0.209
Yes	9 (37.5)	12 (23.5)		
No	15 (62.5)	39 (76.5)		
Death (*n*, %)			0.029	0.865
Yes	8 (33.3)	16 (31.4)		
No	16 (66.7)	35 (68.6)		
Relapse mortality (*n*, %)			2.291	0.130
Yes	8 (33.3)	9 (17.6)		
No	16 (66.7)	42 (82.4)		
Non-relapse mortality (*n*, %)			6.517	0.011
Yes	0 (0.0)	7 (13.7)		
No	24 (100.0)	44 (86.3)		
Time interval between disease diagnosis and transplantation (months)	9.5 (2-21.5)	7.7 (3.5-55.3)	NA	NA
Times of chemotherapy before transplantation	6 (3-12)	5 (2-21)	NA	NA
MNC (×10^8^/kg)	9.67 (3.75-53.13)	11.69 (5.18-29.69)	NA	NA
CD34+ cells (×10^6^/kg)	2.95 (1.65-38.82)	5.63 (1.66-12.78)	NA	NA
Time for engraftment of neutrophils (days)	11 (7-17)	12 (8-19)	NA	NA
Time for engraftment of platelets (days)	14 (7-28)	14 (8-26)	NA	NA

HSCT, hematopoietic stem cell; CR1, first complete remission; MNC, mononuclear cells. NA, not applicable.

### Engraftment and GVHD

Hematopoietic reconstitution was observed in all the patients. The median time of neutrophil engraftment was 11 (7–17) days and 12 (8–19) days in the auto-HSCT and allo-HSCT groups, and the median time of platelet recovery was 14 (7–28) days and 14 (8–26) days, respectively. In the allo-HSCT group, the incidence of grade II-IV acute GVHD (aGVHD) was 13.7% (7/51), and the incidence of localized and extensive chronic GVHD (cGVHD) was 15.7% (8/51) and 5.9% (3/51), respectively. The incidence of aGVHD in the AD group was significantly higher than that in the MSD group (P=0.028). However, there was no significant difference in the incidence of grade II-IV aGVHD (P=0.466).

### Prognosis and survival analyses

**Figure 1:**
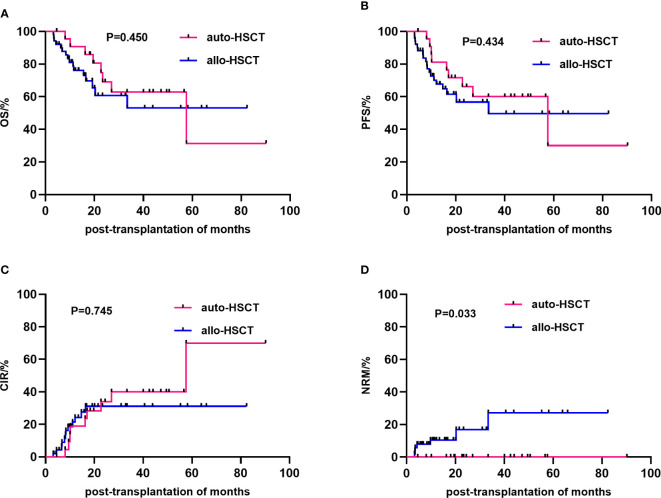
Comparison of the efficacy of different transplantation methods. The overall survival **(A)**, progression-free survival **(B)**, cumulative incidence of relapse **(C)** and non-relapse mortality **(D)** between the allo-HSCT and auto-HSCT groups. HSCT, Hematopoietic stem cell transplantation.

The median follow-up time for all patients was 16.5 (3.17–90.17) months. The 3-year OS rates after transplantation were 62.8 ± 11.4% and 53.0 ± 10.4% (P=0.919), the 3-year PFS rates were 60.1 ± 11.1% and 49.6 ± 9.9% (P=0.434), and the 3-year CIR were 39.9 ± 11.1 and 31.1 ± 7.7(P=0.745) in the auto-HSCT group and allo-HSCT group, respectively (**Table 2**). At the end of the follow-up, 37.5% (9/24) and 23.5% (12/51) (P=0.209) of the auto-HSCT and allo-HSCT groups relapsed, respectively, and 33.3% (8/24) and 31.4% (16/51) (P=0.865) of the patients died after transplantation, respectively. The NRM rate was significantly higher in the allo-HSCT group than in the auto-HSCT group (13.7% vs. 0%, P=0.011) (**Table 1**; **Figure 1**). In the auto-HSCT group, eight patients died of relapse. In the allo-HSCT group, 9 patients died of relapse (MSD=5, AD=4), 4 patients died of severe infection (MSD=1, AD=3), 2 patients died of multiple organ failure (AD group), and 1 patient died of serious aGVHD (AD group).

**Table 2:** Univariate survival analyses of 75 patients(N=75).

Risk factors	3-year Cumulative OS		3-year Cumulative PFS		3-year CIR		3-year Cumulative NRM	
	OS (%, mean ± SD)	*P* value	PFS (%, mean ± SD)	*P* value	CIR (%, mean ± SD)	*P* value	NRM (%, mean ± SD)	*P* value
Type of transplantation		0.450		0.434		0.745		0.033
Allo-HSCT	53.0 ± 10.4		49.6 ± 9.9		31.1 ± 7.7		27.2 ± 11.7	
Auto-HSCT	62.8 ± 11.4		60.1 ± 11.1		39.9 ± 11.1		0	
Source of donors		0.929		0.949		0.697		0.212
Auto-donor	62.8 ± 11.4		60.0 ± 11.1		39.9 ± 11.1		0	
MSD	64.4 ± 12.2		59.0 ± 12.6		32.6 ± 11.2		11.1 ± 10.5	
Source of donors		0.226		0.247		0.958		0.021
Auto-donor	62.8 ± 11.4		60.0 ± 11.1		39.9 ± 11.1		0	
URD	61.2 ± 11.7		56.7 ± 11.6		26.7 ± 11.4		21.5 ± 9.7	
Source of donors		0.164		0.145*		0.808		0.003
Auto-donor	62.8 ± 11.4		60.0 ± 11.1		39.9 ± 11.1		0	
HPD	60.0 ± 25.3*		60.0 ± 18.4*		33.3 ± 19.2*		10.0 ± 9.5*	
Gender		0.019		0.033		0.056		0.345
Male	49.9 ± 8.6		46.8 ± 8.2		44.9 ± 8.7		14.1 ± 6.0	
Female	80.0 ± 12.6		77.0 ± 12.0		13.3 ± 8.8		11.1 ± 10.5	
Age (years)		0.078		0.167		0.246		0.473
<24	42.8 ± 11.8		45.8 ± 11.3		38.9 ± 9.4		24.6 ± 14.5	
≥24	67.1 ± 9.5		59.3 ± 9.6		33.6 ± 9.6		10.4 ± 6.1	
IPI scores		0.139		0.433		0.885		0.185
0-2	63.5 ± 11.5		55.7 ± 10.8		40.9 ± 11.2		5.5 ± 3.8	
≥3	48.9 ± 9.6		49.6 ± 9.6		35.0 ± 9.0		76.5 ± 10.2	
B symptoms		0.615		0.625		0.769		0.137
Yes	50.1 ± 13.8		51.8 ± 12.9		27.3 ± 10.4		28.2 ± 14.5	
No	59.2 ± 8.8		53.7 ± 8.7		40.7 ± 8.8		9.2 ± 5.8	
Disease status at HSCT		0.022		0.008		0.027		0.179
CR1	68.7 ± 7.8		64.1 ± 7.7		28.6 ± 7.5		10.1 ± 5.3	
Non-CR1	28.1 ± 12.7		24.1 ± 11.8		62.9 ± 14.1		34.4 ± 19.9	
Invasion of the mediastinum		0.023		0.006		0.005		0.535
Yes	46.9 ± 9.3		39.4 ± 8.7		53.2 ± 9.5		14.9 ± 6.9	
No	72.8 ± 11.4		75.2 ± 10.9		12.0 ± 6.5		14.5 ± 10.6	
Invasion of the bone marrow		0.775		0.757		0.863		0.760
Yes	61.2 ± 8.5		57.0 ± 8.2		32.9 ± 7.7		14.4 ± 7.0	
No	50.6 ± 13.6		48.6 ± 13.1		39.0 ± 11.8		20.3 ± 15.0	
Conditioning regimen		0.026		0.118		0.123		0.654
TBI-based	48.7 ± 8.5		48.9 ± 8.2		43.5 ± 8.6		13.1 ± 5.9	
Non-TBI-based	77.9 ± 13.1		64.7 ± 13.1		20.3 ± 9.5		18.6 ± 13.2	

*censored.

OS, overall survival; PFS, progression-free survival; CIR, cumulative incidence of relapse; NRM, non-relapse mortality; HSCT, hematopoietic stem cell; CR1, first complete remission; MSD, matched sibling donor; URD, unrelated donor; HPD, haploidentical donor.

**Figure 2:**
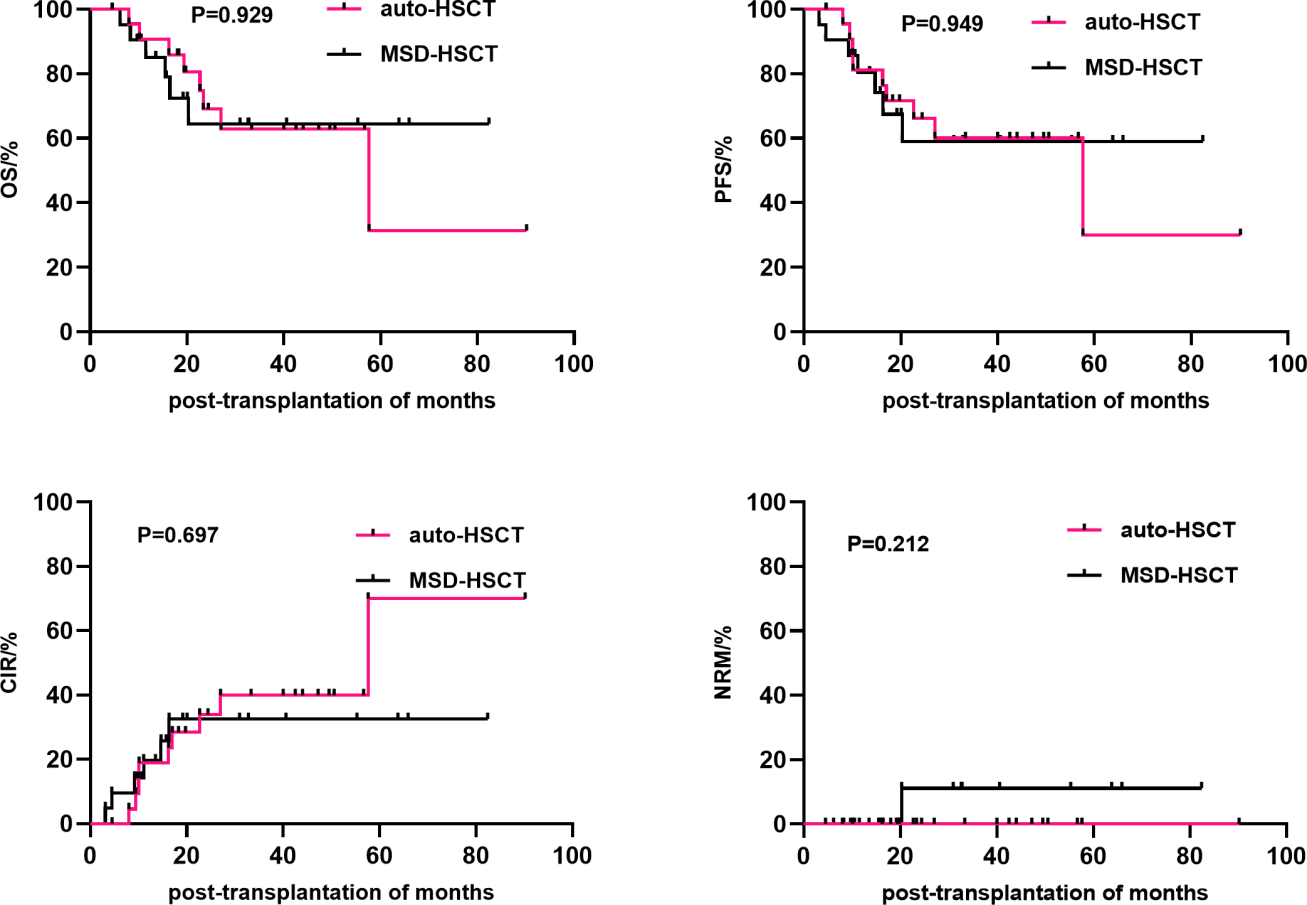
Comparison of the efficacy between auto-HSCT and MSD-HSCT groups. HSCT, Hematopoietic stem cell transplantation.

Univariate analysis showed that gender (P=0.019), disease status at HSCT (P=0.022), mediastinal invasion (P=0.023), and conditioning regimen (P=0.026) were important factors affecting the 3-year cumulative OS. Meanwhile, the significant influencing factors of 3-year cumulative PFS were gender (P=0.033), disease status at HSCT (P=0.008), and mediastinal invasion (P=0.006). For the 3-year CIR, the significant influencing factors were disease status at HSCT (P=0.027) and mediastinal invasion (P=0.005). The type of transplantation (P=0.033) was a risk factor affecting the 3-year cumulative NRM. Additionally, comparing the sources of different donors, the NRM in the URD and HPD groups was significantly higher than that in the auto-HSCT group (P=0.021 and P=0.003, respectively), while there was no significant difference between MSD-HSCT and auto-HSCT. Compared with the MSD-HSCT group, the auto-HSCT group showed an increasing trend in 3-year CIR (39.9 ± 11.1% vs. 32.6 ± 11.2%, P=0.697) and a lower trend in 3-year OS (62.8 ± 11.4% vs. 64.4 ± 12.2%, P=0.929) (**Table 2**; **Figure 2**). The gender, age, IPI scores, disease status at HSCT, invasion of the mediastinum, conditioning regimen, and chemotherapy cycle were important factors affecting the 3-year OS in the allo-HSCT group. The gender, age, IPI scores, disease status at HSCT, invasion of the mediastinum, chemotherapy cycle, and Epstein-Barr virus (EBV) infection were significant factors affecting the 3-year PFS. Age, disease status at HSCT, invasion of the mediastinum, conditioning regimen, and chemotherapy cycle were important factors affecting 3-year CIR. The disease status at HSCT and hemorrhagic cystitis (HC) were significant factors affecting 3-year cumulative NRM in the allo-HSCT group (**Table 2**).

Multivariate analysis showed that CR1 was an independent influencing factor for improving OS (HR=2.498, 95% CI 1.097–5.689, P=0.029) and PFS (HR=2.576, 95% CI 1.196–5.545, P=0.016) in patients who received HSCT. The absence of mediastinal invasion was an independent influencing factor for improving PFS (HR=2.977, 95% CI 1.116–7.943, P=0.029) and decreasing CIR (HR=4.040, 95% CI 1.173–13.920, P=0.027) (**Table 3**) in the patients receiving HSCT. The disease status at HSCT and invasion of the mediastinum were independent risk factors for 3-year OS, PFS and CIR. EBV infection also was an independent influencing factor for 3-year PFS (**Table 3**).

**Table 3:** Multivariate survival analyses of 75 patients.

	Risk factors	*P* value	*HR*(95%*CI*)
OS	Gender	0.133	0.309 (0.067-1.431)
	Disease status at HSCT	0.029	2.498 (1.097-5.689)
	Invasion of the mediastinum	0.093	2.352 (0.867-6.375)
	Conditioning regimen	0.160	2.479 (0.698-8.808)
PFS	Gender	0.056	0.301 (0.088-1.032)
	Disease status at HSCT	0.016	2.576 (1.196-5.545)
	Invasion of the mediastinum	0.029	2.977 (1.116-7.943)
CIR	Gender	0.090	0.271 (0.060-1.224)
	Disease status at HSCT	0.050	2.444 (1.002-5.962)
	Invasion of the mediastinum	0.027	4.040 (1.173-13.920)

OS, overall survival; PFS, progression free survival; CIR, cumulative incidence of relapse.

## Discussion

HSCT is an important treatment modality for T-LBL, especially for refractory and relapsed patients. However, there are limited treatment options, and HSCT has become the last option for patients. Relapse during the intensive phase and second-line treatment without HSCT are risk factors for poor prognosis (20). However, there is still no consensus regarding the transplantation method and donor source. Autologous or allogeneic HSCT after consolidation therapy may improve OS (21, 22). However, the benefit of HSCT in T-LBL and the patient populations who may benefit from HSCT are still controversial. The most favorable method for improving prognosis needs to be selected according to the patient’s condition and disease risk stratification (23).

Research has shown that allo-HSCT should be preferred to conventional chemotherapy as a post-remission treatment for adults with lymphoblastic lymphoma (24). Haploidentical peripheral blood HSCT is safe and effective in T-LBL treatment, with a 3-year OS of up to 70% (25). The NHL-BFM Study Group also reported that allo-HSCT is the only good prognostic factor for patients with relapsed LBL (26). The occurrence of aGVHD may be associated with better outcomes in patients with relapsed/refractory LBL who undergo allogeneic transplantation (27). Moreover, achieving CR1 before allo-HSCT is associated with a favorable OS, regardless of the disease subtype (28). In our study, CR1 was also shown to be an independent risk factor for 3-year OS (P=0.029), PFS (P=0.016) in T-LBL patients. The prognosis of those who underwent allo-HSCT was similar to or slightly worse than that of patients who underwent auto-HSCT.

Some studies have also shown that auto-HSCT is a reasonable option for chemotherapy-sensitive T-LBL patients in CR1. The 5-year OS and event-free survival (EFS) rates were 64% and 47% for the initially treated patients, respectively, and were both 20% for relapsed patients (29). The results vary across studies, and a treatment strategy for adults with chemosensitive T-LBL that includes planned consolidation with HSCT in the first response produces favorable long-term outcomes. For patients who received auto-HSCT, the 4-year EFS rate was 69%. Bone marrow involvement is a significant prognostic factor for worse survival (P=0.02) (21). Won et al. reported a 2-year EFS rate of 50.5% in 13 patients who underwent auto-HSCT for LBL after high-dose chemotherapy (HDC). The status at transplantation was the most predictive factor for survival after HDC/PBSCT (EFS for CR 70.8 ± 9.5% vs. non-CR 20.0 ± 17.9%, P=0.008). Transplantation-related complications were minimal, and infection was the most prevalent complication (30).

HDC/PBSCT is safe for patients with recurrent or refractory pediatric NHL and could replace conventional chemotherapy (30). There are also prospective clinical trial studies showing that tandem auto-HSCT is the optimal treatment strategy for T-LBL in adults. The 3-year progression/relapse rate of the tandem auto-HSCT group was significantly lower than that of the single auto-HSCT and chemotherapy groups (26.5%, 53.1%, and 54.8%, respectively). Further, the 3-year PFS and OS rates of the tandem auto-HSCT group (73.5% and 76.3%, respectively) were significantly higher than those of the single auto-HSCT group (46.9% and 58.3%, respectively) and the chemotherapy group (45.1% and 57.1%, respectively) (31). Some studies have shown that secondary auto-HSCT salvage therapy is also feasible in chemotherapy-sensitive patients who relapse after the first transplantation. The 3-year PFS was 36% (95% CI, 21%-52%), and treatment-related mortality was lower than that reported for allogeneic transplant in this setting. Secondary auto-HSCT should be considered for patients with relapsed NHL after the first transplantation without alternative allogeneic stem cell transplant options (32, 33). In general, auto-SCT showed a trend for improved PFS, whereas no difference in OS was observed between the two arms (34). At present, there is no consensus on the role of auto-HSCT in the treatment of LBL. However, early consolidation therapy after intensive chemotherapy, auto-HSCT, and local radiotherapy can help patients achieve a higher sustained remission rate (35).

There is no established standard for selecting between auto-HSCT and allo-HSCT, but a comprehensive consideration is needed depending on the patient’s condition. Our study also showed that CR1 at transplantation is an important factor affecting patient prognosis. Hazar et al. evaluated 21 patients with LBL (5 and 16 patients with B-LBL and T-LBL, respectively) undergoing HSCT. Among them, 16 patients received allo-HSCT, while 5 patients received auto-HSCT, and the overall OS and EFS were 48% and 44%, respectively (36). In a retrospective multicenter study of the largest series of LBL patients treated with auto-HSCT or MSD-HSCT, MSD-HSCT was associated with fewer relapses than was auto-HSCT (5-year rate, 34% vs. 56%; P=0.004) but was also associated with higher TRM (6-month rate, 18% vs. 3%; P=0.002), which obscured any potential survival benefit (22). Similar data have been reported by other authors (37). Another study showed that MSD-HSCT and auto-HSCT had similar early bone marrow relapse rates (32% vs. 46%, P=0.05), but significantly lower relapse rates at 1 and 5 years were observed with MSD-HSCT (34% vs. 56%, P=0.004). Meanwhile, there were no significant differences in the 5-year PFS (36% vs. 39%, P=0.82) and 5-year OS (44% vs. 39%, P=0.47), and bone marrow involvement at transplantation and non-CR1 were associated with poorer patient prognosis (22).

T-cell LBL often presents as a large mediastinal mass. This location is also a common site of relapse (13, 38). Similar results were observed in our study. There were no significant differences in the 3-year CIR (32.6% vs. 39.9%, P=0.697), 3-year PFS (59.0% vs. 60.0%, P=0.949), and 3-year OS (64.4% vs. 62.8%, P=0.929). Mediastinal invasion at transplantation and non-CR1 were important factors affecting PFS and CIR, and non-CR1 was also an important factor affecting OS. Meanwhile, mediastinal invasion was not an independent risk factor for OS. Similarly, some studies have also shown that CR1 is also a risk factor affecting the efficacy of allogeneic transplantation in T-ALL/LBL patients (28, 39). Moreover, in patients younger than 25 years of age, treatment with ALL intensive regimens up to CR1 followed by allo-HSCT resulted in a 5-year OS of 57% and a treatment-related mortality of only 10% (40). In multicenter study of 24 adult T-LBL patients who underwent HSCT after remission with hyper-CVAD chemotherapy, there was no difference in survival outcomes between the 16 patients with auto-HSCT and the 8 patients with allo-HSCT (41).

In our study, univariate analysis showed that male patients had worse prognosis than did female patients with respect to both 3-year OS (49.9% vs. 80.0%, P=0.019) and 3-year PFS (46.8% vs. 77.0%, P=0.033), consistent with the results of the COG clinical trial. Male patients remained to have a slightly worse outcome than female patients in COG trials, despite the extra year of treatment (42–44). However, multivariate analysis showed that gender was not an independent risk factor of worse survival. This may be related to the higher incidence of disease in men. In addition, univariate survival analysis also showed that TBI-based conditioning regimen was an unfavorable factor affecting 3-year OS (P=0.026), but multivariate survival analysis showed that it was not an independent risk factor (P=0.160). In contrast, some studies have suggested that a TBI-based myeloablative conditioning regimen is an important factor favoring the efficacy of allogeneic transplantation in ALL patients (39). This may be related to various factors such as different stages of the patient’s disease, general condition, and pre-transplantation chemotherapy regimen, which need to be further validated in large-scale, multicenter prospective clinical trials.

## Conclusion

Our study shows that HSCT is an effective consolidation method for the treatment of T-LBL, especially in patients with CR1 and no mediastinal invasion at the time of transplantation. For CR1 patients, auto-HSCT and MSD-HSCT are effective strategies to improve survival, and auto-HSCT has similar to efficacy to MSD-HSCT. However, because MSD-HSCT has a GVL effect, it may be more conducive for long-term survival and reducing relapse, but attention should be paid to the treatment of transplantation-related complications. For patients who are non-CR1, allo-HSCT is the only modality to reduce relapse and improve prognosis, especially MSD-HSCT can greatly improve the survival of patients, followed by matched URD-HSCT and HPD-HSCT.

## Data availability statement

The original contributions presented in the study are included in the article/supplementary material. Further inquiries can be directed to the corresponding authors.

## Ethics statement

Ethical review and approval was not required for the study on human participants in accordance with the local legislation and institutional requirements. Written informed consent to participate in this study was provided by the participants’ legal guardian/next of kin.

## Author contributions

ZL and BZ wrote the manuscript, collected the related literature, and prepared the figures and tables. XF, RG, FY, JW, YZ, KZ, YYL, YFL and JD collected the data. JZ, and YS revised and edited the manuscript. All authors reviewed and approved the final manuscript.
